# Dietary Protein Level in Late Gestation: Effects on Nutritional and Developmental Responses in Jennies and Their Foals

**DOI:** 10.3390/ani16060929

**Published:** 2026-03-16

**Authors:** Yongmei Guo, Jiarong Li, Yajun Shen, Sumei Yan, Binlin Shi, Yanli Zhao, Xiaoyu Guo

**Affiliations:** Key Laboratory of Animal Nutrition and Feed Science at Universities of Inner Mongolia Autonomous Region, Inner Mongolia Agricultural University, Hohhot 010018, China; ymguo2015@163.com (Y.G.);

**Keywords:** jennies in late gestation, dietary protein level, nutrient digestion, plasma amino acids, serum biochemical indexes, donkey foal

## Abstract

This study evaluated the effects of dietary protein levels during late gestation on nutrient digestibility, plasma amino acid profiles in jennies, and donkey foal growth performance. Twenty-four pregnant jennies were randomly assigned to one of three diets with different crude protein (CP) contents during late gestation: 12.48% (HP), 11.52% (MP), and 10.54% (LP) on a dry matter basis. All animals received the same diet immediately after parturition for a duration of 30 days. During the trial, two digestion experiments were conducted, blood samples were collected at 28 and 7 days prepartum, and weekly weight measurements of jennies and foals were recorded. The results indicated that the dietary protein level did not significantly affect feed intake in late gestation. However, apparent nutrient digestibility of dry matter (DM), neutral detergent fiber (NDF), acid detergent fiber (ADF), crude protein (CP), and ether extract (EE), and calcium (Ca) and phosphorus (P) was generally higher in the MP and LP groups than in the HP group, with MP showing the most consistent improvements across nutrients and timepoints (*p* < 0.05). Although the HP diet increased plasma concentrations of certain amino acids, including glutamic acid (Glu), valine (Val), methionine (Met), leucine (Leu), essential amino acids (EAAs), functional amino acids (FAAs), and branched chain amino acids (BCAAs), and elevated serum levels of glucose (GLU), blood urea nitrogen (BUN), and creatinine (CRE), it failed to improve postpartum weight recovery in jennies, highlighting that weight dynamics during this period are governed by factors beyond dietary protein content alone. Specifically, the LP group exhibited significantly higher cumulative postpartum weight loss over weeks 1–4 than the HP group (*p* = 0.004). Regarding offspring performance, both HP and MP diets improved foal birth weight, weekly body weight up to 4 weeks, average daily gain, and body height compared to the LP group (*p* < 0.05), with no significant differences observed between the HP and MP groups. In conclusion, for jennies under the current confined feeding system, a late-gestation diet containing 11.52% CP was adequate to support higher nutrient digestibility in the jennies and better growth performance in their foals, compared to a lower protein level (10.54% CP). However, increasing the dietary CP to 12.48% provided no additional benefits in nutrient utilization or overall productivity.

## Simple Summary

This study investigated the effects of dietary protein levels on body weight changes during late gestation, nutrient digestion, serum parameters in jennies, and foal growth and development. The results indicate that a moderate protein level provides optimal balance: it enhances nutrient digestion, maintains healthy serum parameters, assists jennies in retaining better body condition postpartum, and promotes increased foal birth weight and growth development. These findings hold significant theoretical and practical implications for improving protein nutrition in gestating donkeys, enhancing jennies’ productivity and foal growth performance, thereby boosting profitability in donkey breeding and rearing operations.

## 1. Introduction

Donkeys are used for meat and milk production, and to obtain skin gelatine used in Asian traditional medicines. Donkey skin is the main material for making donkey gelatin, which has the functions of tonifying blood, hemostasis, anti-fatigue, beautifying [1], and alleviating physical fatigue and improving exercise endurance in mice [2]. In comparison with beef, pork, and lamb, donkey meat contains higher essential amino acids, low cholesterol, a low proportion of fatty acids to total fatty acids, a high proportion of polyunsaturated fatty acids, and higher content of iron, VA and VE [3]. Donkey milk generally contains lysozyme, lactoferrin and other bioactive components [4].

At present, there is little research on the nutritional requirements of donkeys in the world. Liang et al. [5] and Li [6] reported that increasing the protein content increases the dry matter (DM), protein and milk fat content of lactating donkey milk. Yin [7] and Zhou [8] reported that the optimal dietary energy level can increase the daily weight gain of gestating jennies, thereby supporting maternal body condition maintenance and fetal development, and significantly affect the growth and fattening speed and feed conversion rate of fattened donkeys. The medium energy group (digestible energy (DE) levels of 10.49 MJ/kg) and the high energy group (DE levels of 10.92 MJ/kg) significantly increased the fat content and fat deposition in fattening donkeys [8].

Dietary protein is the major factor influencing animal growth and metabolism. However, research into the protein requirements of the gravid female is mainly focused on mammals such as cattle, sheep and pigs. Wu et al. [9] and Yu et al. [10] showed that dietary protein levels had a significant effect on pregnant females and young stock. Liang et al. pointed out that the dietary protein level in late gestation had a significant effect on the content of DM, milk protein, aspartic acid (Asp), alanine (Ala), tyrosine (Tyr), arginine (Arg), phenylalanine (Phe), valine (Val), isoleucine (Ile), leucine (Leu), histidine (His), essential amino acids (EAAs), non-essential amino acids (NEAAs), flavor amino acids (DAAs) and branched chain amino acids (BCAAs) in milk [11]. How the protein nutrition level in late gestation affects the nutrient digestion of the jennies and the birth weight of the foal is still not clear. Thus, the objective of this study was to investigate the effects of dietary protein level in late gestation on nutrient digestion, blood amino acid content, serum biochemical indexes, postpartum weight change in Jennies and growth and development of donkey foals to provide a basis for the formulation of protein nutritional requirements in late donkey gestation.

## 2. Materials and Methods

The experiment was conducted at a donkey farm in Helinger (a county in Hohhot, Inner Mongolia, China). All animal procedures were performed in accordance with the experimental procedures at Inner Mongolia Agricultural University ([2019]035), which were approved by the Animal Ethics and Welfare Committee on 26 December 2019.

### 2.1. Animals, Design and Management

In a completely randomized experimental design, 24 female Dezhou donkeys [7.15 ± 1.69 years old, 270.11 ± 63.24 kg body weight, 1.63 ± 0.48 parity, and 58.66 ± 4.28 days pre-delivery (corresponding to 301.34 ± 4.28 days of gestation, based on an average gestation length of 360 days)] were randomly assigned to one of three dietary treatments (*n* = 8 donkeys per treatment). Each donkey was considered an experimental unit. The diets varied in crude protein (CP) content on an air-dry basis: high crude protein (HP; 12.48% CP), medium crude protein (MP; 11.52% CP), and low crude protein (LP; 10.54% CP). The dietary trial began when the jennies were approximately 58.66 ± 4.28 days prior to expected parturition (around day 300 of gestation). The total experiment lasted 90 days, comprising a 10-day adaptation period, followed by an 80-day feeding trial that spanned from late gestation through early lactation. Specifically, the jennies received the experimental diets for approximately 50 days prepartum and continued on the same diets for 30 days postpartum. The diets were formulated with a concentrate-to-forage ratio of 30:70. The forage component was chopped, and the concentrate was a pelleted mixture formulated specifically for this study. Throughout the trial, the Jennies were individually housed with their foals in single stalls and fed twice daily at 07:00 and 14:00, with water available ad libitum. Jennies were housed individually in indoor pens with concrete floors bedded with straw. Each pen was equipped with an individual feeder and an automatic water nipple. All pens had partial outdoor access for natural light and ventilation. The amount of feed offered was adjusted weekly based on the previous week’s intake to maintain a targeted refusal rate of approximately 5% of the amount offered, ensuring ad libitum intake while minimizing selective refusals. Representative feed samples were collected weekly and subsampled for chemical analysis. Orts samples were collected from each animal during digestibility trials for nutrient analysis. The ingredients and nutritional composition of the experimental diets are detailed in Table 1.

### 2.2. Sample Collection and Chemical Analysis

Throughout the entire trial, the daily feed intake of gestating donkeys was recorded, and the average daily intake was calculated every two weeks. Apparent nutrient digestibility of DM, CP, ether extract (EE), neutral detergent fiber (NDF), acid detergent fiber (ADF), calcium (Ca), and phosphorus (P) was assessed using the endogenous indicator method, with 4N hydrochloric acid-insoluble ash (AIA) as the marker. Fecal samples were collected from days 26 to 30 and from days 49 to 53, at 8:00 a.m., 11:30 a.m., 3:00 p.m., and 7:30 p.m., with 40 g collected per time point. Samples from each donkey were pooled, thoroughly mixed, and stored at −20 °C for subsequent nutrient analysis. Digestibility was calculated using the following formula:

Apparent nutrient digestibility (%) = 100 − (Nutrient content in feces ÷ Nutrient content in feed) × (Feed AIA content ÷ Fecal AIA content) × 100.

Blood samples were collected from the jugular vein of all experimental donkeys before morning feeding at two time points: 4 weeks before delivery (approximately day 332 of gestation) and 1 week before delivery (approximately day 353 of gestation). Serum was separated and stored at −20 °C for biochemical analysis. Concentrations of essential amino acids (EAAs), non-essential amino acids (NEAAs), total amino acids (TAAs), DAAs, functional amino acids (FAAs), and branched-chain amino acids (BCAAs) in serum were quantified using an L-8900 automatic amino acid analyzer (Hitachi High-Tech Corporation, Tokyo, Japan) according to the method described in GB/T 5009.124-2003 [12].

Serum levels of albumin (ALB), triglycerides (TG), total protein (TP), creatinine (CRE), glucose (GLU), and blood urea nitrogen (BUN) were determined using an automatic biochemical analyzer (Hitachi 7020, Hitachi, Ltd., Tokyo, Japan), with assay kits provided by LePu Diagnostics (Beijing, China). Serum concentrations of high-density lipoprotein cholesterol (HDL-C) and low-density lipoprotein cholesterol (LDL-C) were measured using a microplate reader with colorimetric assay kits supplied by Nanjing Jiancheng Bioengineering Institute (Nanjing, China).

Each gestating donkey was weighed immediately after delivery and subsequently once daily in the morning before feeding until the fourth week postpartum. Postpartum weight loss proportions during 1–2 weeks, 2–4 weeks, and 1–4 weeks were calculated as follows:

Postpartum weight loss ratio = (End weight of the period − Initial weight of the period) ÷ Initial weight of the period × 100%.

Birth weight and body size of donkey foals were recorded immediately after birth. Indicators of body size consisted of body length, body height, chest girth and tube girth, and were measured weekly until the fourth week. Body length was the distance from the anterior margin of the scapula to the end of the sciatic bone; body height was the vertical distance from the top of the withers to the ground; heart girth was the distance around the rib cage directly behind the forelimb; tube girth was the circumference of the finest part of the left forelimb cannon bone.

### 2.3. Statistical Analysis

All statistical analyses were performed using SAS version 9.2 (SAS Institute Inc., Cary, NC, USA). Data on nutrient digestibility and plasma amino acid concentrations were analyzed using one-way analysis of variance (ANOVA) with dietary treatment as the fixed effect. When a significant treatment effect was detected (*p* ≤ 0.05), Duncan’s multiple range test was used for post hoc comparisons. Feed intake, serum biochemical indexes, weight loss of jennies, body weight, daily gain and body size of donkey foals were analyzed in a complete randomized design model for repeated measures using the Proc Mixed procedure in SAS. The Mixed model included fixed effects for dietary protein level, sampling time and their interactions, and the random effects of the animal. Data was presented as mean and standard error of means (SEM). Statistical significance was declared at *p* ≤ 0.05.

## 3. Results

### 3.1. Feed Intake of Jennies

As shown in Table 2, dietary protein level had no significant effect on donkey feed intake at any stage of late gestation or across the whole experimental period (*p* > 0.05).

### 3.2. Nutrient Digestibility of Jennies

As shown in Table 3, dietary protein level significantly influenced nutrient digestibility during late gestation. At four weeks prepartum, the MP group demonstrated significantly higher digestibility of DM, CP, ADF, and P than the HP group (*p* < 0.05). Compared with the LP group, the MP group also exhibited greater digestibility of DM, CP and ADF (*p* < 0.05), whereas the LP group surpassed the HP group in DM and ADF digestibility (*p* < 0.05). Additionally, Ca digestibility was higher in both the MP and LP groups than in the HP group (*p* < 0.05).

At one week prepartum, the MP group showed significantly higher digestibility of all measured nutrients relative to the HP group (*p* < 0.05). The MP group also displayed greater digestibility of CP, EE, and P compared with the LP group (*p* < 0.05). Meanwhile, the LP group had higher digestibility of DM, NDF, ADF, and Ca than the HP group (*p* < 0.05).

### 3.3. Plasma Amino Acid Profile of Jennies

As shown in Table 4, dietary protein level significantly influenced the plasma amino acid profile during the 4 weeks prepartum. Glycine (Gly) concentration was significantly higher in the LP and MP groups than in the HP group (*p* < 0.05). In contrast, concentrations of Val, Met, and TAA were significantly higher in the HP group compared to both the MP and LP groups (*p* < 0.05). The HP group also showed numerically higher concentrations of EAA and FAA than the LP group, though these differences did not reach statistical significance (*p* = 0.085 and *p* = 0.093, respectively). No significant differences were observed among the three groups for the remaining amino acid parameters (*p* > 0.05).

The effects of dietary protein became more pronounced at 1 week prepartum (Table 5). Compared with the MP and LP groups, the HP group had significantly higher concentrations of Glu, Leu, BCAA, and higher EAA/TAA and EAA/NEAA ratios (*p* < 0.05), with no significant differences observed between the MP and LP groups. The HP group showed an upward trend in EAA compared with the LP group (*p* = 0.068). Furthermore, increasing dietary protein level showed a trend toward elevated concentrations of threonine (Thr), serine (Ser), Val, Arg, NEAA, TAA, and DAA, although these differences did not reach statistical significance (*p* > 0.05).

### 3.4. Serum Biochemical Indexes of Jennies

As shown in Table 6, dietary protein level in late gestation significantly affected serum concentrations of GLU and BUN in donkeys. The HP group had significantly higher GLU levels than the LP group (*p* = 0.035), while the MP group showed intermediate values with no significant difference from either group. The BUN content in the HP group was significantly higher than that in both the MP and LP groups (*p* = 0.027), which themselves did not differ. No significant differences were observed among groups in ALB, TP, TG, CRE, HDL-C, or LDL-C (*p* > 0.05).

### 3.5. Postpartum Weight Loss of Jennies

As shown in Table 7, dietary protein level in late gestation did not significantly affect the body weight of Jennies during weeks 1–2 or weeks 3–4 postpartum (*p* = 0.083 and *p* = 0.053, respectively). However, it significantly influenced cumulative weight loss over weeks 1–4 postpartum (*p* = 0.004). The LP group exhibited a higher weight loss ratio than the HP group, while the MP group showed no significant difference compared with either the HP or LP groups.

### 3.6. The Growth and Development of Donkey Foals

As shown in Table 8, maternal dietary protein level during late gestation significantly influenced foal body weight. From birth to week 1 and week 4 postpartum, body weights in the HP group were consistently and significantly higher than those in the LP group (*p* < 0.05), with no significant difference observed between the HP and MP groups.

Average daily gain increased with maternal dietary protein level (Table 9). Both the HP and MP groups exhibited significantly higher daily gains during weeks 1–2, weeks 3–4, and cumulatively over weeks 1–4, compared to the LP group (*p* < 0.05), while no significant difference was found between the HP and MP groups.

As presented in Table 10, the HP and MP groups showed significantly greater body height than the LP group (*p* < 0.05), with no significant difference between the two higher protein groups. However, maternal late-gestation dietary protein level did not significantly affect foal body length, chest girth, or tube girth.

## 4. Discussion

Protein is a crucial nutrient governing animal growth, reproduction, and lactation performance. Its requirement varies with body weight, age, production stage, and environmental conditions, and both deficiency and excess can adversely affect health. To date, research on the effects of dietary protein levels on nutrient digestion and metabolism in gestating dams has mainly focused on species such as cattle, sheep, and pigs, with inconsistent findings. Relevant studies in donkeys remain scarce. Therefore, this study systematically investigated the effects of different dietary protein levels on nutrient digestion, serum metabolism, postpartum recovery, and foal growth in late-gestation donkeys, aiming to establish a theoretical basis for precise protein nutrition during gestation.

### 4.1. Effects of Dietary Protein Level in Late Gestation on Feed Intake in Jennies

The results of this study indicate that dietary protein levels had no significant effect on feed intake of jennies during late gestation. This observation is consistent with previous findings that different dietary protein levels did not significantly affect feed intake in fattening donkeys [13]. In gestating jennies, nutrient partitioning prioritizes fetal development and the maintenance of maternal reserves, whereas in fattening donkeys, nutrient utilization is primarily directed toward weight gain. Therefore, although the feed intake response appeared similar between the two physiological states in this study, the underlying mechanisms governing these responses warrant further investigation. Yin et al. [14] also observed no significant effect of dietary protein level on DM intake in peripartum dairy cows. These findings align with the results of the present study. However, Gaillard et al. found that dairy cows fed a high-protein diet (16% CP) had higher feed intake compared to those fed a low-protein diet (14% CP) [15]. This discrepancy likely reflects species-specific physiological demands: lactating cows are highly sensitive to dietary protein due to their amino acid requirements for milk production, whereas the non-lactating jennies in this study had lower metabolic protein demands. Hossain et al. also reported significantly higher feed intake in broilers fed a high-protein diet compared to those fed medium- or low-protein diets [16]. However, the absence of such an effect in the gestating jennies of the current study underscores the modulating role of physiological status, in this case, pregnancy and its associated metabolic adaptations, in the intake response to dietary protein concentration. Another study found that feed intake was significantly higher in a low-protein group than in a high-protein group for Yanshan cashmere goats [11]. In summary, while the response of feed intake to dietary protein level can be influenced by animal species, physiological stage, and diet composition, the preponderance of evidence—including the present study—suggests that moderate variations in dietary protein do not consistently affect voluntary feed intake.

### 4.2. Effects of Dietary Protein Level in Late Gestation on Nutrient Digestibility in Jennies

Apparent nutrient digestibility directly reflects an animal’s efficiency in utilizing dietary components. The present study showed a similar trend, where digestibility first increased and then decreased with increasing protein level. The MP group (11.52% CP) exhibited the best performance, with significantly higher digestibility of DM, CP, EE, Ca, and P compared to the HP group (12.48% CP). Wang et al. found in fattening donkeys that digestibility initially increased and then decreased with rising dietary protein levels, peaking in the medium-protein group [13]. Zhou [17] reported in donkey foals that the digestibility of CP and EE increased significantly with increasing dietary protein levels, while DM and CF digestibility were not significantly affected. Although these studies were conducted in different physiological stages, they collectively demonstrate that protein level is a key regulator of nutrient digestibility in donkeys. In the absence of extensive research specifically in late-gestating jennies, these findings provide a valuable reference framework for the current study, suggesting that the observed quadratic response in digestibility may be a common biological phenomenon across donkey categories. Wang et al. [18] also indicated that increasing dietary DE and CP levels led to a significant linear and quadratic increase in digestible Ca utilization in late-gestating Yili mares. These collective results suggest that appropriately increasing dietary protein levels can improve nutrient digestibility. However, the quadratic response observed indicates the presence of an optimal level, beyond which digestibility plateaus or declines. This may be due to the moderate elevation of protein levels, potentially promoting the secretion and activity of intestinal proteases, thereby enhancing amino acid absorption. Further investigation into the molecular regulatory mechanisms underlying this effect warrants consideration.

### 4.3. Effects of Dietary Protein Level in Late Gestation on Plasma Amino Acid Profiles and Serum Biochemical Indices in Jennies

Blood parameters are important indicators reflecting protein metabolism and nutritional homeostasis. Plasma amino acids serve as crucial indicators reflecting animal nutritional intake, physiological status, and production performance, playing a key role in maintaining organismal health and metabolic balance [19]. Especially during gestation, amino acids act as essential precursors for the synthesis of proteins, hormones, and signaling molecules, exerting a significant influence on fetal development. A deficiency in amino acids can markedly impede fetal protein biosynthesis [20]. Plasma amino acids, as one of the primary components of blood, are considered the main form of amino acid reserves in animals and play a crucial role in maintaining the body’s amino acid balance [21]. Several studies have confirmed that feeding sows diets with high amino acid levels during late gestation can enhance daily weight gain in piglets [22], reduce BUN levels in low-protein groups [23], and potentially promote fetal growth by modulating nitrogen and lipid metabolism [5]. Although this observation in swine cannot be directly extrapolated to equids, it highlights the importance of maternal amino acid supply during late gestation for offspring growth [24]. In sows, the metabolic pathway involves the direct transfer of dietary amino acids to fetal circulation via placental transporters, with minimal interference from hindgut microbial protein synthesis [25]. In contrast, equids possess functional ceca and colons where microbial fermentation can generate additional amino acids. However, absorption of these microbially derived amino acids is minimal, exerting no significant impact on the body’s overall amino acid nutritional status [26]. Nevertheless, this local synthesis is primarily considered a reflection of microbial metabolic activity that may benefit the host by supporting gut health- a hypothesis that warrants further investigation. Liang et al. demonstrated that feeding medium- and high-protein diets significantly increased the content of DM, various amino acids, and EAA in donkey milk [5]. Chi et al. further found that high-protein diets positively regulated the plasma levels of certain amino acids (e.g., aspartic acid, threonine) in donkey foals [27]. In this study, analysis of the plasma EAA/NEAA and EAA/TAA ratios revealed that as dietary protein levels increased, the concentrations of plasma glutamate, leucine, EAA, FAA, and BCAA all showed varying degrees of elevation, accompanied by synchronous increases in the EAA/TAA and EAA/NEAA ratios. These results indicate that elevating dietary protein levels during late gestation helps optimize the plasma amino acid balance in donkeys, enhances the enrichment of functional AA, and thereby provides a nutritional foundation for improving maternal health and offspring development.

Serum biochemical parameters, such as TP and GLU, serve as indicators of an animal‘s metabolic status and tissue health [28]. In this experiment, serum GLU and BUN levels were significantly elevated in the HP group. Glucose serves as the primary energy source for the uterus, and maintaining adequate blood glucose levels is crucial for meeting the surging energy demands during parturition [29,30]. The significant increase in serum glucose in donkeys fed the HP diet suggests a metabolic shift that may be associated with increased protein intake. Although the specific mechanisms cannot be determined from the current data, this observation aligns with previous findings in equids where high-protein diets influenced glucose metabolism [31]. Further studies directly measuring gluconeogenic activity or glucose turnover are needed to elucidate the underlying pathways. Concurrently, BUN and CRE levels directly reflect the intensity of amino acid catabolism. Elevated levels observed in this study are consistent with increased dietary protein intake and subsequent deamination of amino acids [32]. These findings indicate altered nitrogen metabolism in response to the high-protein diet. The observed increase in BUN with increasing dietary protein levels corroborates the findings of Wei et al. in finishing pigs [33], although it is important to note that direct comparisons between swine and equids should be made cautiously due to differences in hindgut digestive physiology. The long-term consumption of high-protein diets by late-gestation donkeys may have implications for nitrogen utilization and feed costs, warranting further investigation into the optimal protein balance for this physiological stage.

### 4.4. Effects of Dietary Protein Level in Late Gestation on Postpartum Weight Loss in Jennies and Foals’ Growth and Development

Late gestation is a critical period characterized by rapid fetal development and surging nutrient demand. Adequate nutrient supply from the dam is essential not only to support fetal growth but also to lay the foundation for successful subsequent lactation. The nutritional demands during late gestation are well-documented in livestock species such as swine and sheep, where nutrient intake has been shown to significantly influence maternal and offspring outcomes. Studies have shown that reducing the dietary protein level of lactating sows significantly decreases their weaning weight, milk yield, and piglet average daily gain (ADG) [34]. Zhang [35] also reported that feeding a low-nutrient diet during gestation significantly reduced the peripartum and weaning weights of primiparous sows, while improved nutritional levels tended to increase lactational weight loss. Zhao et al. [36] found that supplemental concentrate grazing during late gestation and early lactation significantly reduced postpartum weight loss in Sunit ewes. These findings raise the question of whether similar responses occur in donkeys. In the present study, higher dietary protein levels were associated with reduced postpartum weight loss in jennies, with the LP group showing the greatest loss. To further explore this relationship, we calculated the protein-to-energy (P:E) ratios of the experimental diets. The ratios were 43.6, 46.7, and 50.8 g CP/Mcal DE for the LP, MP, and HP groups, respectively. For comparison, the recommended P:E ratio for gestating mares is 40–50 g CP/Mcal DE [37]. The LP diet fell near the lower boundary of this range, while the HP diet slightly exceeded it. This observed association suggests that insufficient protein intake relative to energy—reflected by a lower P:E ratio—before parturition may be linked to greater postpartum weight loss. Conversely, higher P:E ratios appeared to mitigate this loss, possibly by providing a better balance of substrates for maternal tissue maintenance and milk production. Further research measuring changes in energy balance or body composition would be needed to confirm the underlying mechanisms.

Regarding offspring growth, the present study found that foals born to jennies fed MP (11.52% CP) and HP (12.48% CP) diets had higher birth weights, average daily gains, and body heights compared to those from the LP group. These findings are consistent with some previous reports—for instance, Hatfield et al. [38] observed that lambs born to ewes fed a high-protein diet (14.9% CP) had higher birth weights than those from a low-protein group (11.3% CP). However, other studies have reported contrasting results, indicating that responses may vary by species and experimental conditions. One possible explanation for the improved foal growth observed in the MP and HP groups is enhanced milk quality, as Liang et al. [5] previously reported that feeding medium- and high-protein diets to donkeys during late gestation increased the content of dry matter, milk protein, and most amino acids in postpartum milk. Consistently, Glade et al. [39] reported in mares that soybean meal supplementation during early lactation increased milk protein content and absolute amino acid concentrations, thereby improving foal growth performance. These findings in a closely related equid species further support the likelihood that improved milk composition mediates the growth benefits observed in the present study. Although milk parameters were not directly measured in the present study, this reported effect on milk composition offers a plausible hypothesis for the superior foal growth observed here. Future studies directly measuring milk yield and composition are needed to confirm this link.

In summary, the present data demonstrate that increasing dietary protein level during late gestation is associated with reduced postpartum weight loss in jennies and improved early growth in their foals. These observed outcomes provide a basis for recommending an optimal protein level (11.52% CP) for late-gestation jennies, while the physiological mechanisms underlying these effects require further study incorporating direct measurements of energy balance, nitrogen metabolism, and milk composition. This study systematically elucidates the physiological and productive responses of late-gestating donkeys to dietary protein levels, providing an important reference for formulating protein nutrition standards during donkey gestation. Future research could further explore the relationships between protein levels and lactation performance, long-term foal development, and dam reproductive intervals to refine whole-cycle nutritional management strategies.

## 5. Conclusions

In conclusion, this study establishes a protein requirement benchmark for late-gestating donkeys, demonstrating that 11.52% CP optimizes the trade-off between maternal metabolic demands and foal development. While higher protein intake (12.48% CP) offers no additional benefits and may impose metabolic costs, the identified moderate level supports efficient nutrient utilization, minimizes postpartum weight loss, and promotes early foal growth. These findings provide an evidence-based reference for formulating gestation diets in donkeys, thereby improving reproductive efficiency and offspring quality in donkey production systems where empirical data have previously been lacking.

## Figures and Tables

**Table 1:** The composition and nutrient levels of HP, MP, and LP groups (air-dry basis, %).

Items	Prenatal Diet			Postpartum Diet
	HP	MP	LP	
Dietary composition (%)				
Millet straw	54.00	54.00	54.00	52.08
Alfalfa	16.00	16.00	16.00	15.92
Corn	19.08	18.03	16.23	19.87
Soybean meal	6.00	7.71	7.80	7.75
Corn gluten meal	0.60	1.50	3.30	1.58
Corn germ meal	0.60	0.69	0.60	0.47
Wheat bran	2.25	0.60	0.60	0.47
NaCl	0.37	0.37	0.37	0.40
CaCO_3_	0.33	0.33	0.33	0.35
CaHPO_4_	0.66	0.66	0.66	0.70
Premix ^(1)^	0.10	0.10	0.10	0.40
Total	100.00	100.00	100.00	100.00
Nutrient level (%)				
Moisture	12.77	12.84	12.91	9.19
DE (MJ/kg) ^(2)^	10.27	10.27	10.23	10.35
DM	87.23	87.16	87.09	90.81
CP	12.48	11.52	10.54	12.22
EE	2.60	2.57	2.59	2.67
Ash	6.67	6.68	6.65	6.33
NDF	46.73	46.77	47.20	46.09
ADF	27.53	27.50	27.51	27.33
Ca	0.72	0.72	0.72	1.09
P	0.33	0.33	0.33	0.33

^(1)^ One kg of premix provided the following: VA 6,000,000 IU, VD 1,250,000 IU, VE 15,000 IU, Fe 20 g, Cu 8.0 g, Zn 60.0 g, Mn 60 g, Mn 360 mg, Se 300 mg, Co 500 mg. ^(2)^ DE was a calculated value, while the others were measured values. DM = dry matter; CP = crude protein; EE = ether extract; NDF = neutral detergent fibre; ADF = acid detergent fibre; Ca = calcium; P = phosphorus.

**Table 2:** Effect of Dietary Protein Level in Late Gestation on Donkey Feed Intake (kg/day·head).

Items	HP	MP	LP	SEM	*p*-Value
8 weeks before delivery	4.99	5.40	5.80	0.402	0.405
6 weeks before delivery	5.64	5.49	5.84	0.261	0.649
4 weeks before delivery	5.38	5.11	5.71	0.317	0.451
2 weeks before delivery	5.38	5.11	5.95	0.332	0.245
Whole experiment period	5.30	5.27	5.38	0.328	0.256

SEM = standard error of least square means; HP: high crude protein; MP: medium crude protein; LP: low crude protein.

**Table 3:** Effect of Dietary Protein Level during Late Gestation on Nutrient Digestibility in Jennies (%).

Items ^(1)^	HP ^(2)^	MP	LP	SEM	*p*-Value
4 weeks before delivery					
DM	58.74 ^c^	68.86 ^a^	62.39 ^b^	0.554	0.020
CP	76.46 ^b^	78.55 ^a^	76.58 ^b^	1.259	0.048
EE	65.14	72.34	68.85	1.682	0.090
NDF	75.07	78.43	80.23	1.134	0.058
ADF	66.33 ^c^	71.93 ^a^	68.95 ^b^	0.583	<0.001
Ca	55.56 ^b^	68.66 ^a^	68.26 ^a^	1.986	0.002
P	61.75 ^b^	70.26 ^a^	68.79 ^ab^	1.908	0.045
1 week before delivery					
DM	78.27 ^b^	80.26 ^a^	80.36 ^a^	0.475	<0.001
CP	74.54 ^c^	77.89 ^a^	75.85 ^b^	0.250	<0.001
EE	58.56 ^b^	67.01 ^a^	63.51 ^b^	1.550	0.003
NDF	71.45 ^b^	76.78 ^a^	75.40 ^a^	0.620	<0.001
ADF	67.20 ^b^	71.84 ^a^	71.01 ^a^	0.370	<0.001
Ca	62.66 ^b^	69.23 ^a^	68.25 ^a^	1.160	<0.001
P	56.51 ^c^	66.69 ^a^	62.75 ^b^	0.900	<0.001

^(1)^ DM = dry matter; CP = crude protein; EE = ether extract; NDF = neutral detergent fibre; ADF = acid detergent fibre; Ca = calcium; P = phosphorus. HP: high crude protein; MP: medium crude protein; LP: low crude protein. ^(2)^ abc means within the same row, not followed by the same letters, are significantly different. Differences with *p* ≤ 0.05 were considered significant.

**Table 4:** Effect of Dietary Protein Level in Late Gestation on Plasma Amino Acid Profile (μmol/L) during the 4 weeks prepartum.

Items ^(1)^	HP	MP	LP	SEM	*p*-Value
Asp	39.77	36.70	35.08	0.000	0.248
Thr	116.48	119.13	116.44	8.077	0.981
Ser	118.05	122.20	116.59	6.872	0.908
Glu	151.56	125.46	121.56	19.870	0.060
Gly	159.28 ^b^	178.37 ^a^	185.22 ^a^	14.275	0.029
Ala	92.44	102.21	99.68	15.664	0.602
Cys	6.17	5.18	6.57	0.900	0.405
Val	103.63 ^a^	94.05 ^b^	85.58 ^b^	3.425	0.025
Met	16.01 ^a^	13.54 ^b^	13.10 ^b^	1.326	0.036
Ile	33.01	28.02	26.42	9.311	0.504
Leu	82.10	64.36	61.24	7.363	0.287
Tyr	37.63	27.05	25.27	9.323	0.105
Phe	31.33	27.56	27.15	7.975	0.659
Lys	45.19	43.75	40.11	4.291	0.795
His	52.40	47.39	46.99	8.892	0.569
Arg	14.27	23.04	17.01	11.143	0.465
Pro	67.39	58.48	60.02	8.122	0.213
EAA	489.78	446.55	418.44	12.356	0.085
NEAA	672.29	646.11	660.28	21.124	0.789
TAA	1157.18 ^a^	1092.70 ^b^	1031.66 ^b^	22.341	0.025
DAA	479.50	485.21	478.28	13.132	0.949
FAA	251.98	208.38	192.88	14.735	0.093
BCAA	121.34	89.78	82.46	12.324	0.197
EAA/TAA	0.43	0.41	0.40	0.016	0.557
EAA/NEAA	0.75	0.68	0.67	0.050	0.675

^(1)^ Asp = aspartic acid; Thr = threonine; Ser = serine; Glu = glutamic acid; Ala = alanin; Cys = Cysteine; Val = valine; Met = methionine; Ile = isoleucine; Leu = leucine; Tyr = tyrosine; Phe = phenylalanine; Lys = lysine; His = histidine; Arg = arginine; Pro = proline; EAA = essential amino acids; NEAA = non-essential amino acids; TAA = total amino acids; DAA = flavor amino acids; FAA = functional amino acids; BCAA = branched chain amino acids; EAA/TAA = essential amino acids/total amino acids; EAA/NEAA = essential amino acids/non-essential amino acids. HP: high crude protein; MP: medium crude protein; LP: low crude protein. ab means within the same row, not followed by the same letters, are significantly different. Differences with *p* ≤ 0.05 were considered significant.

**Table 5:** Effect of Dietary Protein Level in Late Gestation on Plasma Amino Acid Profile (μmol/L) during the one week prepartum.

Items ^(1)^	HP	MP	LP	SEM	*p*-Value
Asp	32.06	36.83	33.09	2.528	0.572
Thr	144.98	118.93	109.12	9.115	0.101
Ser	117.60	112.96	105.84	7.323	0.800
Glu	157.33 ^a^	123.25 ^b^	125.32 ^b^	5.939	0.010
Gly	177.34	183.82	172.18	6.408	0.583
Ala	90.96	99.00	95.72	5.588	0.727
Cys	6.12	5.85	5.90	0.359	0.920
Val	110.20	99.32	96.23	6.220	0.560
Met	13.68	13.96	14.03	1.161	0.984
Ile	45.24	29.19	30.55	4.105	0.085
Leu	105.24 ^a^	59.15 ^b^	59.63 ^b^	8.023	0.009
Tyr	36.75	26.20	29.58	3.156	0.199
Phe	32.68	26.83	29.93	2.865	0.538
Lys	60.48	47.43	52.73	5.254	0.403
His	46.26	46.64	45.10	2.530	0.933
Arg	24.68	18.29	17.35	2.852	0.305
Pro	64.53	57.79	61.51	2.898	0.470
EAA	601.05	458.47	460.88	35.852	0.068
NEAA	676.28	662.67	649.01	22.848	0.787
TAA	1277.33	1121.14	1109.88	55.070	0.192
DAA	495.76	481.04	463.58	13.237	0.388
FAA	287.25 ^a^	202.29 ^b^	200.69 ^b^	12.927	0.002
BCAA	153.07 ^a^	92.23 ^b^	90.33 ^b^	11.924	0.016
EAA/TAA	0.47 ^a^	0.41 ^b^	0.41 ^b^	0.012	0.024
EAA/NEAA	0.89 ^a^	0.70 ^b^	0.69 ^b^	0.040	0.016

^(1)^ Asp = aspartic acid; Thr = threonine; Ser = serine; Glu = glutamic acid; Ala = alanin; Cys = cysteine; Val = valine; Met = methionine; Ile = isoleucine; Leu = leucine; Tyr = tyrosine; Phe = phenylalanine; Lys = Lysine; His = histidine; Arg = arginine; Pro = proline; EAA = essential amino acids; NEAA = non-essential amino acids; TAA = total amino acids; DAA = flavor amino acids; FAA = functional amino acids; BCAA = branched chain amino acids; EAA/TAA = essential amino acids/total amino acids; EAA/NEAA = essential amino acids/non-essential amino acids. HP: high crude protein; MP: medium crude protein; LP: low crude protein. ab means within the same row, not followed by the same letters, are significantly different. Differences with *p* ≤ 0.05 were considered significant.

**Table 6:** Effect of Dietary Protein Level in Late Gestation on Serum Biochemical Indexes of Jennies.

Items ^(1)^	HP Group	MP Group	LP Group	SEM	*p*-Value
GLU (mmol/L)	4.35 ^a^	3.93 ^ab^	3.62 ^b^	0.128	0.035
ALB (g/L)	32.04	30.62	30.04	1.984	0.804
TP (mg/mL)	68.92	82.41	71.47	4.118	0.233
TG (mmol/L)	0.52	0.57	0.51	0.080	0.243
CRE (umol/L)	95.96	91.19	83.28	2.259	0.084
HDL-C (umol/L)	2.26	1.40	1.57	0.251	0.161
LDL-C (umol/L)	1.36	1.21	1.22	0.074	0.478
BUN (mg/L)	154.61 ^a^	128.87 ^b^	121.90 ^b^	6.386	0.027

^(1)^ GLU = glucose; ALB = albumin; TP = total protein; TG = triglycerides; CRE = creatinine; HDL-C = high-density lipoprotein cholesterol; LDL-C = low-density lipoprotein cholesterol; BUN = blood urea nitrogen. HP: high crude protein; MP: medium crude protein; LP: low crude protein. ab means within the same row, not followed by the same letters, are significantly different. Differences with *p* ≤ 0.05 were considered significant.

**Table 7:** Effect of Dietary Protein Level during Late Gestation on Postpartum Weight Loss Ratio in Jennies (%).

Items ^(1)^	HP	MP	LP	SEM	*p*-Value
Week 1–2	−1.89	−2.81	−3.37	0.548	0.083
Week 3–4	−2.05	−2.51	−4.38	0.673	0.053
Week 1–4	−3.91 ^a^	−4.51 ^ab^	−7.61 ^b^	0.750	0.004

^(1)^ HP: high crude protein; MP: medium crude protein; LP: low crude protein. ab means within the same row, not followed by the same letters, are significantly different. Differences with *p* ≤ 0.05 were considered significant.

**Table 8:** Effect of Maternal Dietary Protein Level in Late Gestation on Body Weight of Donkey Foals (kg).

Items ^(1)^	HP	MP	LP	SEM	*p*-Value
newborn	30.20 ^a^	31.80 ^a^	26.60 ^b^	0.895	0.016
Week 1	32.76 ^a^	33.50 ^a^	29.70 ^b^	1.253	0.025
Week 2	37.16	38.76	33.13	0.525	0.056
Week 3	40.20 ^a^	41.20 ^a^	37.25 ^b^	1.081	0.048
Week 4	44.40	45.77	39.10	1.025	0.053

^(1)^ HP: high crude protein; MP: medium crude protein; LP: low crude protein. ab means within the same row, not followed by the same letters, are significantly different. Differences with *p* ≤ 0.05 were considered significant.

**Table 9:** Effect of Maternal Dietary Protein Level in Late Gestation on Daily Gain in Donkey Foals (kg).

Items ^(1)^	HP	MP	LP	SEM	*p*-Value
Week 1–2	0.51 ^a^	0.52 ^a^	0.45 ^b^	0.003	0.005
Week 2–4	0.52 ^a^	0.50 ^a^	0.43 ^b^	0.028	0.042
Week 1–4	0.51 ^a^	0.50 ^a^	0.45 ^b^	0.049	0.035

^(1)^ HP: high crude protein; MP: medium crude protein; LP: low crude protein. ab means within the same row, not followed by the same letters, are significantly different. Differences with *p* ≤ 0.05 were considered significant.

**Table 10:** Effect of Maternal Dietary Protein Level in Late Gestation on Body Size in Donkey Foals.

Items ^(1)^	HP	MP	LP	SEM	*p*-Value
Body height (cm)					
newborn	91.63 ^a^	92.00 ^a^	87.33 ^b^	1.059	0.045
Week 1	91.85 ^a^	92.32 ^a^	88.21 ^b^	0.425	0.038
Week 2	92.14 ^a^	92.53 ^a^	89.33 ^b^	0.325	0.037
Week 3	92.62 ^a^	93.41 ^a^	90.32 ^b^	0.253	0.059
Week 4	92.89	93.73	90.58	0.023	0.064
Body length (cm)					
newborn	60.00	60.50	60.66	0.128	0.426
Week 1	65.00	66.50	63.20	0.950	0.162
Week 2	65.60	66.00	65.40	1.089	0.958
Week 3	69.50	68.00	68.66	1.188	0.764
Week 4	71.17	70.28	71.46	1.382	0.872
Chest girth (cm)					
newborn	68.50	69.50	69.00	0.725	0.728
Week 1	72.16	74.50	73.60	1.204	0.531
Week 2	73.00	75.00	74.67	0.668	0.135
Week 3	75.00	76.25	77.24	0.996	0.352
Week 4	76.52	78.40	79.00	1.067	0.302
Tube girth (cm)					
newborn	11.10	11.00	11.23	0.189	0.423
Week 1	11.20	11.20	11.22	0.221	0.256
Week 2	11.40	11.33	11.52	0.173	0.457
Week 3	11.50	11.40	11.60	0.244	0.583
Week 4	11.70	11.40	11.80	0.187	0.462

^(1)^ HP: high crude protein; MP: medium crude protein; LP: low crude protein. ab means within the same row, not followed by the same letters, are significantly different. Differences with *p* ≤ 0.05 were considered significant.

## Data Availability

The data from the study is available from the corresponding authors upon reasonable request.

## Abbreviations

The following abbreviations are used in this manuscript:

EAA	Essential Amino Acid
NEAA	Nonessential Amino Acid
DAA	Delicious Amino Acids
BCAA	Branched Chain Amino Acid
CP	Crude Protein
DM	Dry Matter
EE	Ether Extract
NDF	Neutral Washing Fibre
ADF	Acid Washing Fibre
Ca	Calcium
P	Phosphorus
AIA	Acid-insoluble Ash
TAA	Total Amino Acid
FAA	Functional Amino Acid
ALB	Albumin
TG	Triglyceride
TP	Total protein
CRE	Creatinine
GLU	Glucose
BUN	Blood Urea Nitrogen
HDL-C	High-density Lipoprotein Cholesterol
LDL-C	Low-density Lipoprotein Cholesterol
ADG	Average Daily Gain
